# Analysis of the *Ush2a* Gene in Medaka Fish (*Oryzias latipes*)

**DOI:** 10.1371/journal.pone.0074995

**Published:** 2013-09-23

**Authors:** Elena Aller, Ana V. Sánchez-Sánchez, Javier U. Chicote, Gema García-García, Patricia Udaondo, Laura Cavallé, Marina Piquer-Gil, Antonio García-España, Manuel Díaz-Llopis, José M. Millán, José L. Mullor

**Affiliations:** 1 CIBER de Enfermedades Raras (CIBERER), Valencia, Spain; 2 Grupo de Investigación en Enfermedades Neurosensoriales, IIS-La Fe, Valencia, Spain; 3 Unitat de Recerca, Hospital Joan XXIII, Institut de Investigacio Sanitaria Rovira I Virgili (IISPV), Universitat Rovira i Virgili, Tarragona, Spain; 4 Servicio de Oftalmología, Hospital Universitario La Fe, Valencia, Spain; 5 Servicio de Otorrinolaringología, Hospital Universitario La Fe, Valencia, Spain; 6 Unidad de Genética y Diagnóstico Prenatal, Hospital Universitario La Fe, Valencia, Spain; 7 Bionos, SL, Biopolo La Fe – Hospital La Fe, Valencia, Spain; Institut Curie, France

## Abstract

Patients suffering from Usher syndrome (USH) exhibit sensorineural hearing loss, retinitis pigmentosa (RP) and, in some cases, vestibular dysfunction. USH is the most common genetic disorder affecting hearing and vision and is included in a group of hereditary pathologies associated with defects in ciliary function known as ciliopathies. This syndrome is clinically classified into three types: USH1, USH2 and USH3. USH2 accounts for well over one-half of all Usher cases and mutations in the *USH2A* gene are responsible for the majority of USH2 cases, but also for atypical Usher syndrome and recessive non-syndromic RP. Because medaka fish (*Oryzias latypes*) is an attractive model organism for genetic-based studies in biomedical research, we investigated the expression and function of the *USH2A* ortholog in this teleost species. *Ol-Ush2a* encodes a protein of 5.445 aa codons, containing the same motif arrangement as the human USH2A. *Ol-Ush2a* is expressed during early stages of medaka fish development and persists into adulthood. Temporal *Ol-Ush2a* expression analysis using whole mount in situ hybridization (WMISH) on embryos at different embryonic stages showed restricted expression to otoliths and retina, suggesting that *Ol-Ush2a* might play a conserved role in the development and/or maintenance of retinal photoreceptors and cochlear hair cells. Knockdown of *Ol-Ush2a* in medaka fish caused embryonic developmental defects (small eyes and heads, otolith malformations and shortened bodies with curved tails) resulting in late embryo lethality. These embryonic defects, observed in our study and in other ciliary disorders, are associated with defective cell movement specifically implicated in left-right (LR) axis determination and planar cell polarity (PCP).

## Introduction

Usher syndrome (USH) is the most frequent genetic cause of combined deafness and blindness causing hearing loss, retinitis pigmentosa (RP) and, in some cases, vestibular dysfunction. USH is associated with defects in ciliary function like Bardet-Biedl syndrome, Joubert syndrome, Senior-Loken syndrome and some forms of non-syndromic RP [1], [2]. Based on clinical features of the hearing impairment, Usher syndrome is classified into three types: I, II, and III (USH1, USH2, USH3). Usher syndrome type I is the most severe form with profound congenital deafness and vestibular dysfunction. USH2 is characterized by moderate non-progressive hearing loss without vestibular dysfunction and USH3 is distinguished from USH1 and USH2 by the progressive nature of its hearing loss [3].

USH2 accounts for well over one-half of all Usher cases and up to date, 3 genes are known to be involved in the pathogenesis of this clinical form: *USH2A*, *GPR98* and *DFNB31* [4], [5], [6], [7]. Mutations in the *USH2A* gene are responsible for the majority of USH2 cases [8], [9], [10] and are also responsible for atypical Usher syndrome and recessive non-syndromic RP [11], [12]. Two main isoforms have been described for this gene. The short isoform_a, reported to be 5 kb, encoding a protein of 170 kDa; and the long isoform_b, that expands the length of coding sequence to 15 kb, encoding a 600 kDa protein [5].

USH2A_isoform_b protein is mostly extracellular except for a membrane-spanning segment followed by an intracellular PDZ-binding domain at the C-terminus. Its function has been related to the true cilia of the retinal photoreceptors and the microvilli of cochlear hair cells. In the inner ear, USH2A_iso_b contributes to the conformation of ankle links, necessary for the development and maintenance of stereocilia cohesion [13]. In the retina, USH2A_iso_b would be part of the complex participating in the delivery of cargo to the outer segment of vertebrate photoreceptor cells [14], [15].

The majority of USH genes have been knocked out in mice. All mutant mice suffer from inner ear defects, but only the *USH2A* knockout mice develop a detectable retinal degeneration [14], [16], [17]. In addition to mouse knock out mutants, zebrafish mutants have also been described for some genes causing Usher syndrome. Zebrafish mutants have been described for 4 USH1 genes: *MYO7A* (*mariner*) [18], *CDH23* (*sputnik*) [19], [20], *PCDH15* (*orbiter*) [21] and *USH1C* [22]. Regarding USH2 genes, Ebermann et al. (2010) [23] used “knock down” models for *USH2A* and *GPR98* in a study focused on the modifier gene *PDZD7*. Additionally, the *GPR98* gene was also characterized in zebrafish by Gibert et al. (2005) [24]. In most of these fish mutants, there is early photoreceptors cell death, which is absent in the majority of murine models, making fish a good model to study the combined eye and ear physiopathology of USH [16].

Small laboratory fish such as zebrafish and medaka, the Japanese killifish, are attractive vertebrate animal models that are easy to handle and are ideally suited for genetic studies because of their large numbers of progeny per generation [25]. Draft genome sequences for both zebrafish and medaka (Medaka Genome Project [http://dolphin.lab.nig.ac.jp/medaka]) are already available. Thus, fish are becoming increasingly important models in biomedical research [26] **.** A compact genome that lacks the complex repetitive elements observed in zebrafish, and the availability of several inbred strains [27] make the medaka fish model especially suited for genome-based analyses. Because the medaka fish is an attractive and economic model organism for genetic-based studies in biomedical research, we analyzed the gene structure, expression and function of human *USH2A* ortholog in this teleost species.

## Materials and Methods

### Animal Strains and Maintenance

This study was carried out in strict accordance with the recommendations contained in the Guide for the Care and Use of Laboratory Animals of the National Institutes of Health. The protocol was approved by the Animal Welfare Ethical Committee (CEBA) of the “Hospital Universitario La Fe”. Adult medaka (*Oryzias latipes*) CAB strain animals were kept in recirculating water aquaria at 28°C on at 14-hr light/10-hr dark dailycycle. Embryos were collected by natural spawning in Yamamoto solution [28] and staged as previously described [29]. Embryos were raised at 25°C.

### Cloning of Medaka Fish *Ush2a* cDNA and Sequencing

Blast searches, using Zebrafish USH2A protein (CAK04893.2) as bait, and intron-exon borders determination were performed as previosuly described [30], [31]. Alignment of protein sequences were done using ClustalW program from the Network Protein Sequence Analysis [32]. The signal peptide sequence was determined with the program SignalP 4.0 Server [33] with a cut-off of 0.650. The domains LamGL, LamNT, EGF-Lam, LamG and FN3 with E-value lower than 1×10^−3^ and the transmembrane domain were found out using the Conserved Domain Search Service (CD Search) from the National Center for Biotechnology Information and the program TMHMM server v2.0 [34], [35]. Finally, the PDZ1-binding domain was located as previously stated [5]. Primers based on cDNA sequence predictions obtained were used to amplify the full-length *Ol-Ush2a* open reading frame from whole larvae and adult eye cDNA (see Table 1 for primer sequences). PCR products were separated and purified using gel electrophoresis and sequenced on an ABI 3500XL analyzer using the fluorescent dideoxy terminator method (Applied Byosistems).

**Table 1:** Primers used to amplify and sequence the full-length open reading frame (ORF) of *Ol-Ush2a*.

NAME	SEQUENCE 5′-3′
**P1D**	ATGGAACCATGACCGTCATCAC
**P1R**	TGCTCTCAGATGTATTCGGAC
**P2D**	AACAGCCCTGGTTGGACTGG
**P2R**	TCCATCCTTAGACCAAACAAGC
**P3D**	GTGTGATCAAAACACT
**P3R**	GAGTCTGGATGGCAGGAAC
**P4D**	TTTGTGTTCCAACACGCCATG
**P4R**	GCAGGTAAAGTCAGTCCTGTG
**P5D**	TACACAGGGTGGAACAATCATC
**P5R**	AACCAGTTTACAGCGGCACTG
**P6D**	CAGAAGCCATGGACTACAGTC
**P6R**	TCAACACAACCCTGAGTGTTAC
**P7D**	CTCGGCACATACTCAGACTG
**P7int_aD**	AATTCCAACGCTCCAGGTCC
**P7int_bD**	TTGTCGACCTTCTTCCGTAC
**P7intR**	AGACCAGCAGGTCGAGATG
**P7R**	TGGTAACAGGAGTTGGGAGAC
**P8D**	ACCCAATGGAGAGGTTCATGG
**P8R**	GAGACCAGGTAGTTGGTACTG
**P9D**	GTTTCTTCCTCACTGTAGAGC
**P9R**	TATCCTTCACAGCACTGGTGC
**P10D**	GACATCCTGCTTCGATCCAG
**P10R**	ACGGTAACGTTCTTGTCCATC
**P11D**	CTACAGCAGTGACAACCAGTG
**P11R**	GGCAATCTTTCAGGTGTAGTC
**P12D**	TCAATGCTGGAAGTCGCAAG
**P12R**	GGCTTGTTTGGTCGACACAG
**P13D**	TCCTTCACAGTGACAGATTTGC
**P13R**	GAAGGCAGTGGAGATATTCTTG
**P14D**	GTGACTGGTCCTCTGTACTC
**P14R**	GTGGGCTCCAAGTAAGTGAC
**P15D**	GGCTGGTTTACAAGAAGCACC
**P15intD**	CCATTGAGAAAGCTTCACACC
**P15R**	CAGCAGTATGAACCACAGTTC
**P16D**	CAGGTGACGTGTACAACAGAC
**P16R**	CTCGTCCTAAAGATGTGTGTC

### RNA Isolation and RT-PCR

Total RNA was isolated from total embryo, larvae and adult tissues using Trizol (Invitrogen, Carlsbad, CA). Reverse transcription was performed according to manufacturer instructions using the Gene Amp Gold RNA Core Kit (Applied Biosystems). Sequence primers used to analyze the spatiotemporal expression pattern of *Ol-Ush2a* were Direct Primer: 5′-AACAGCCCTGGTTGGACTGG and Reverse Primer: TGCTCTCAGATGTATTCGGAC- 3′.

### In Situ Hybridization (ISH)

We performed whole-mount in situ hybridization on medaka fish embryos according to standard procedures [36] using a cRNA probe labeled with digoxigenin-UTP. The probe consisted of nt457–1440of *Ol-Ush2a* mRNA amplified by RT-PCR on cDNA prepared from adult eye using primers DirectCLON: 5′- AAGAATGAATTCGGAAGTGTGCTGTTTCAACC-3′ and ReverseCLON: 5′- AAGAATCTCGAGACAGTTGACTGAGTCTGGTC-3′. The resulting amplified fragment was cloned into the pCS2+ expression vector and sequenced with universal primers (T7 and SP6) for insert sequence checking. The mRNA probe was generated using the T7 promoter. Hybridization was detected with alkaline phosphate-conjugated anti-digoxigenin antibody followed by incubation with nitrobluetetrazolium and BCIP (5-bromo-4-chloro-3-indolyl phosphate). In some experiments, embryos were raised with 0.003% phenylthiourea (PTU) to inhibit pigment formation.

### Morpholino Injections

Antisense morpholinos (GeneTools, Philomath, OR) were injected in CAB embryos at st. 2 using a pressure Narishige IM300 microinjector [30]. Embryos were injected with 0.1–1.00mM of Ol_*ush2a*MO1 (MO1) targeted to the translation start site (5′-TCATCAGCAGCAGCACTGGAGACAT-3′), 0.05–1.00 mM of Ol_ush2aMO2 (MO2) also targeted to the translation start site (5′-TCCAAAGTTTCCTCCTTCTCATGCC-3′), 0.10–1.00 mM of Ol_ush2aMO3 (MO3) targeted to the splice donor site of exon 5 (5′-CTGTCATGGATCTGGAAATCCAAAA-3′) and 1.00 mM of Ol_ush2aMO4 (MO4) targeted to the splice donor site of exon 29 (5′-CATCTGAATTCCTTAATGTGAAGTA-3′), The sequence of control MO for Ol_ush2aMO1 (MO1C) was 5′-TCATgAcCAcCAcCACTcGAGACAT-3′, this sequence correspond to a 5-mispair oligo and it is an specific control for Ol_ush2aMO1. The sequence of control MO (MOC) used for the rest of experiments was 5′-CCTCTTACCTCAGTTACAATTTATA-3′.

### Inmunohistochemistry

For whole-mount imaging of stereocilia and retina, embryos were fixed in ‘BT’ fix (4% PFA, 0.15 mM CaCl2, 4% sucrose in PBS) o/n at 4°C, rinsed four times for 5 minutes each in PBS +0.01% Triton X-100, and then permeabilized with 2% Triton X-100 in PBS o/n at 4°C. They were rinsed four times for 5 minutes each in PBS +0.01% Triton X-100, blocked for 2 hours at room temperature in 10% NGS +0.01% Triton X-100, then incubated with Alexa-Fluor-488–phalloidin (Invitrogen) and *Zpr-1* antibody (The zebrafish international resource Center, Eugene, OR, USA) diluted in block o/n at 4°C. Following four 5-minute PBS +0.01% TritonX-100 washes, embryos were incubated in secondary antibody and TOPRO solution for one hour at room temperature. After rinsing four times for 5 minutes each in PBS-Triton, embryos were mounted using fluoromount G medium. In some experiments, embryos were raised with 0.003% phenylthiourea (PTU) to inhibit pigment formation.

## Results

### *Ol-Ush2a* cDNA and Protein Structure

**Figure 1:**
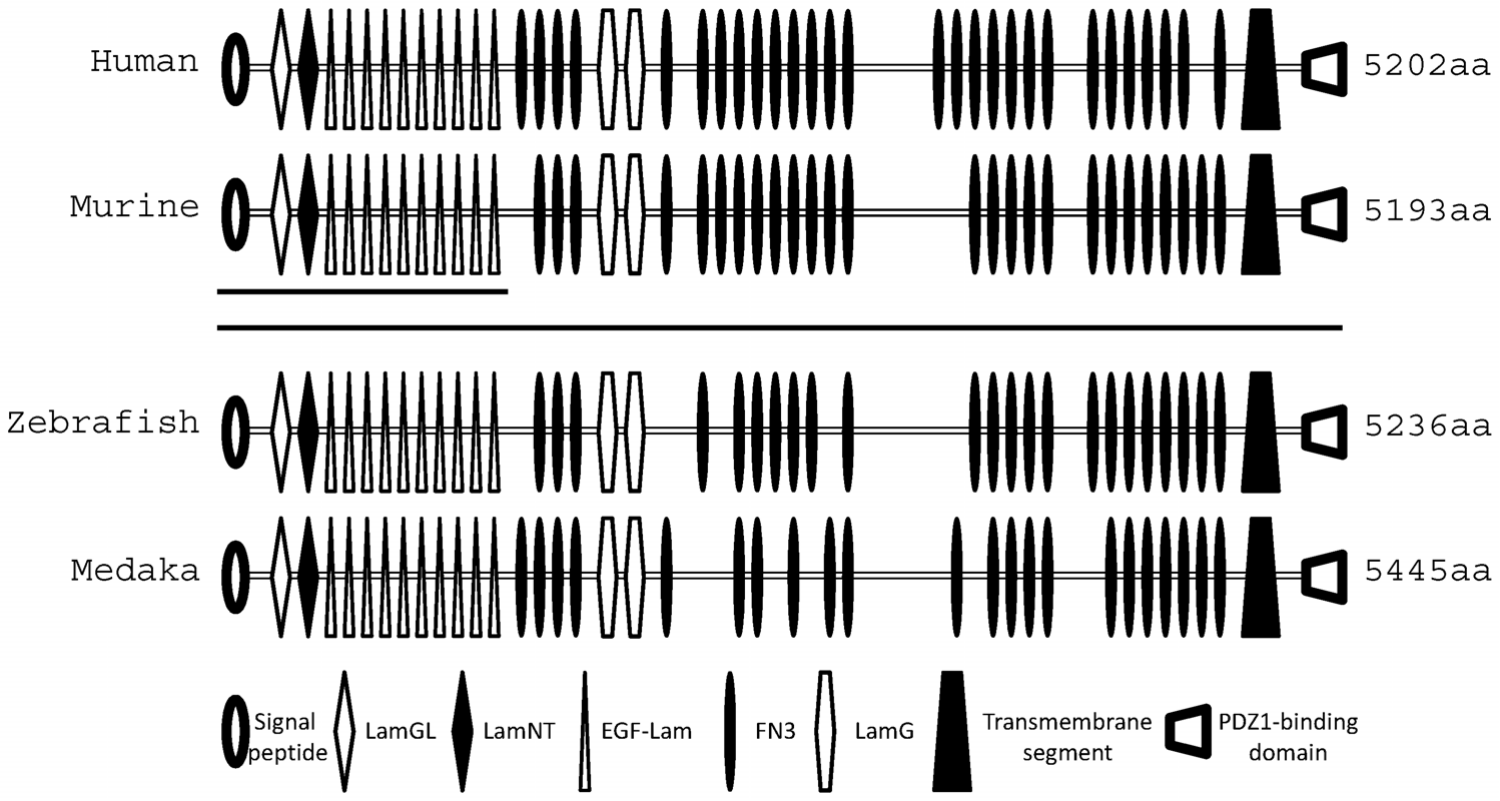
Medaka fish Ol-Ush2a predicted protein. Motif alignment of the human, murine, zebrafish and medaka USH2A proteins. The two lines beneath the murine Ush2a denote the two predicted variants reported (USH2A_iso_a: 170 kDa and USH2A_iso_b: 600 kDa). Symbols representing different motifs are given at the bottom of the diagram: LamGL, LamG-like jellyroll fold domain; EGF-Lam, laminin-type epidermal growth factor-like domain; FN3, fibronectin type 3; LamG, laminin G domain.

Using bioinformatics tools, a single ortholog of human *USH2A* was identified in medaka fish genome at chromosome 3. By PCR amplification of embryonic and ocular cDNA based on these predictions followed by sequencing, the *Ol-Ush2a* gene was characterized. It was assembled from 16 overlapping fragments. This transcript is 16.5 kb in length, with an ORF of 5.445 aa codons (GenBank Accession Number: KF278972). Medaka fish and human predicted proteins have the same motif arrangement, with a large extracellular domain consisting of LamGL, LamNT, EGF-Lam, LamG, and FN3 motif repeats, a single membrane-spanning segmentand a PDZ-binding C terminus predicted to reside intracellularly (Fig. 1).

**Figure 2:**
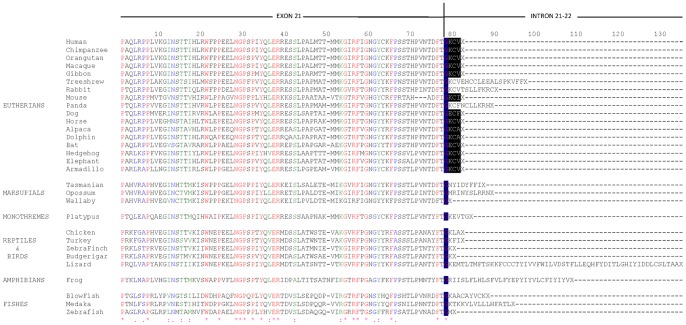
Evolutionary analysis of USH2A isoform_a. Alignment of the exons that correspond to the hypothetic short isoforms of *USH2A.* Only in Primates and most Eutherians the “KCV end” seems to be conserved.

Human *USH2A* has several alternative splice variants. We have investigated the presence of the most frequent of these isoforms (isoform_a) in medaka fish embryos. An evolutionary analysis of Ush2a_iso_a points out that the short isoform would be specific for eutherian species (Fig. 2).

### *Ol-Ush2a* Expression Pattern

**Figure 3:**
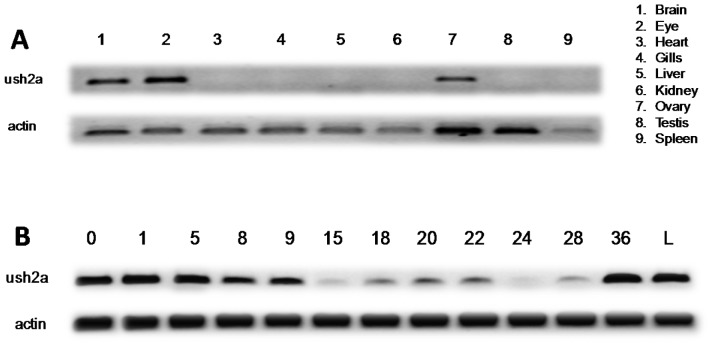
Spatial and temporal *Ol-Ush2a* expression pattern analysis performed by RT-PCR amplification. **A**: RT-PCR amplification from adult organs. **B**: RT-PCR amplification from different embryonic developmental stages.

**Figure 4:**
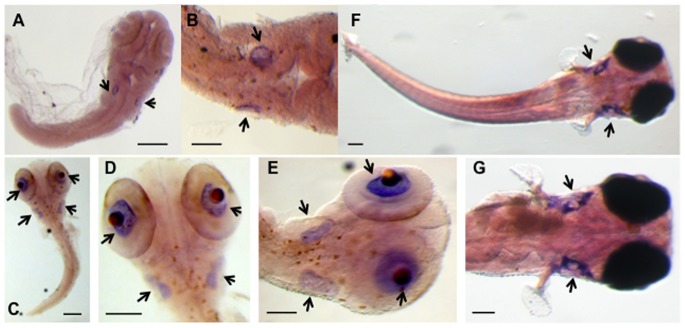
*Ol-Ush2a* expression patern obtained by ISH experiments. **A, B**: Stages 22–23. **C, D, E**: Stage 30, treated with PTU. **F,G**: Stages 36–38. Scale bars: A, C, F and G: 100 µm. B, D and E: 50 µm.

To determine the spatial and temporal *Ol-Ush2a* expression patterns we performed RT-PCR and whole mount *in situ* hybridization analysis of gene expression. *Ol-Ush2a* RT-PCR amplification from adult organs showed *Ol-Ush2a* expression restricted to the brain, eye and ovary (Fig. 3A). When *Ol-Ush2a* expression was analyzed at different embryonic stages, strong *Ol-Ush2a* expression was detected at early developmental stages (st. 0–9). The high *Ol-Ush2a* expression level decreases during gastrulation (st. 15), increases at neurula stage (st. 18) and is maintained during somite formation stages (st. 20–28). During late embryonic stages (st. >30) and after hatching (36) *Ol-Ush2a* expression is upregulated (Fig. 3B). WMISH experiments showed that *Ol-Ush2a* mRNA expression was first detected in the embryonic ear at embryonic stages 22–23, when otic vesicles appear, persisting through all later stages examined (Fig. 4A–G). We also observed *Ol-Ush2a* expression in the retina of embryos treated with PTU at 30–32 developmental stages (Fig. 4C–E).

### Morpholino Knockdown of *Ol-Ush2a* in Medakafish Embryos

In order to analyze the role of *Ol-Ush2a* during early development, we used morpholino oligonucleotides to knock down the function of *Ol-Ush2a*: MO1 and MO2 were designed to block the translation initiation site, MO3 blocked splicing at the intron-4-exon-5 boundary and MO4 blocked splicing at intron-28-exon-29 boundary.

**Figure 5:**
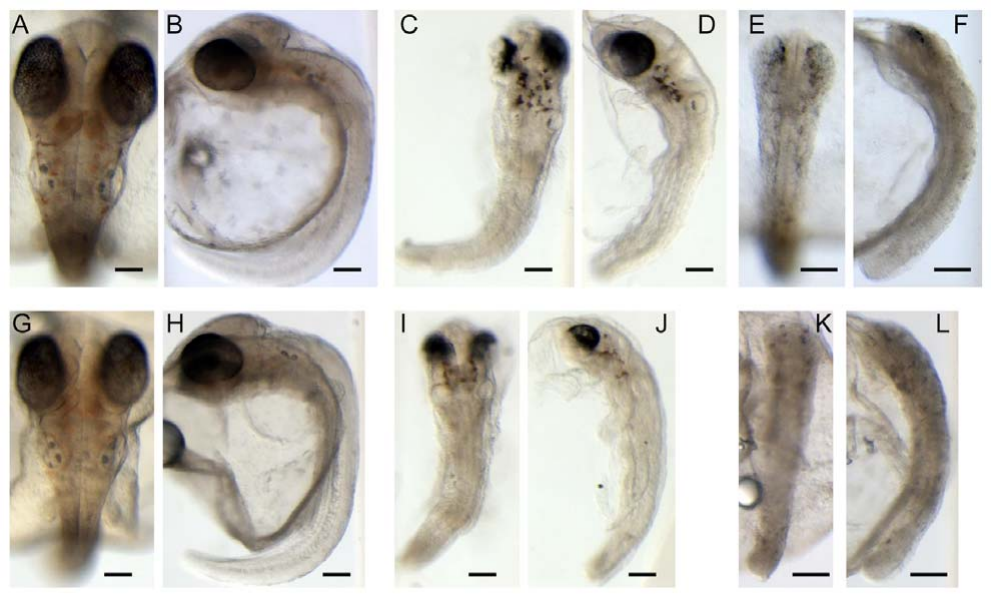
Medaka embryos obtained after MO1 and MO2 injections. **A, B, G, H:**MO1 C (A, B) and MO C (G, H) embryos at 72 hpf. **C, D, I, J:** MO1 (C, D) and MO2 (I, J) injected embryos at 72 hpf, showing a **mild phenotype**. **E, F, K, L:** MO1 (E, F) and MO2 (K, L) injected embryos at 72 hpf, showing a **severe phenotype**. Scale bars: 100 µm. Embryos in frontal (A, C, E, G, I, K) and lateral position (B, D, F, H, J, L).

**Figure 6:**
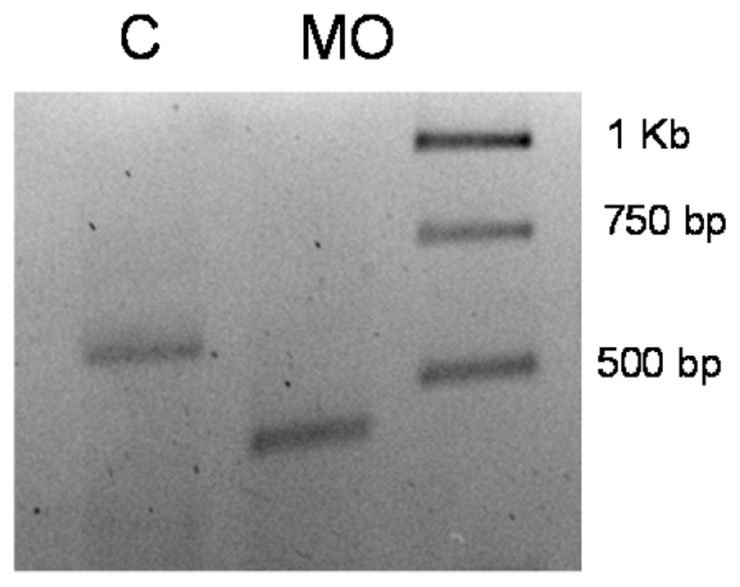
RT-PCR results from total RNA extracted from control and morphant MO3 embryos at 96 hpf. C. Control embryos. Expected size: 573 bp MO. Morphant embryos. Expected size: 444 pb.

Depletion of *Ol-Ush2a* after injection of MO1 or MO2 caused the same phenotype with a delay in embryonic development and evident embryonic malformations (Fig. 5, Table 2). At 72 hpf, MO1 and MO2 injected embryos with less severe phenotypes presented small eyes and heads and otolith malformations (Mild phenotype. Fig. 5C, D, I, J). These embryos were shorter and had curved tails and also presented pigmentation defects. Morphant embryos with more severe phenotypes presented serious generalized malformations (Severe phenotype. Fig. 5E, F, K, L). Not surprisingly MO1 and MO2 injections prevented hatching. Early embryonic lethal phenotypes (at 24 hpf) were obtained with MO3 at concentrations 1-0.5 mM. No effect in embryo development and larvae behavior was observed after injection of MO4 at 1 mM concentration. Using RT-PCR analysis of MO4 injected embryos, we identified an aberrant splice form skipping exon 29, producing an in-frame splicing of exons 28–30 which may explain the lack of phenotype of this morpholino (Fig. 6).

**Table 2:** Phenotypic analysis of *Ol-Ush2a* loss of function.

	normal phenotype %	*Ol-Ush2a*-specific phenotype %	Mortality %	n
*MO C*	92	0	3	356
*MO1 C*	95	0	2	215
*MO1*	3	78	14	324
*MO2*	4	82	11	196
*MO3*	0	0	100	153
*MO4*	94	0	4	124

Nonspecific phenotypes (i.e., injection phenotypes) are not represented in the table, but represent the difference between the reported values and 100%.

**Figure 7:**
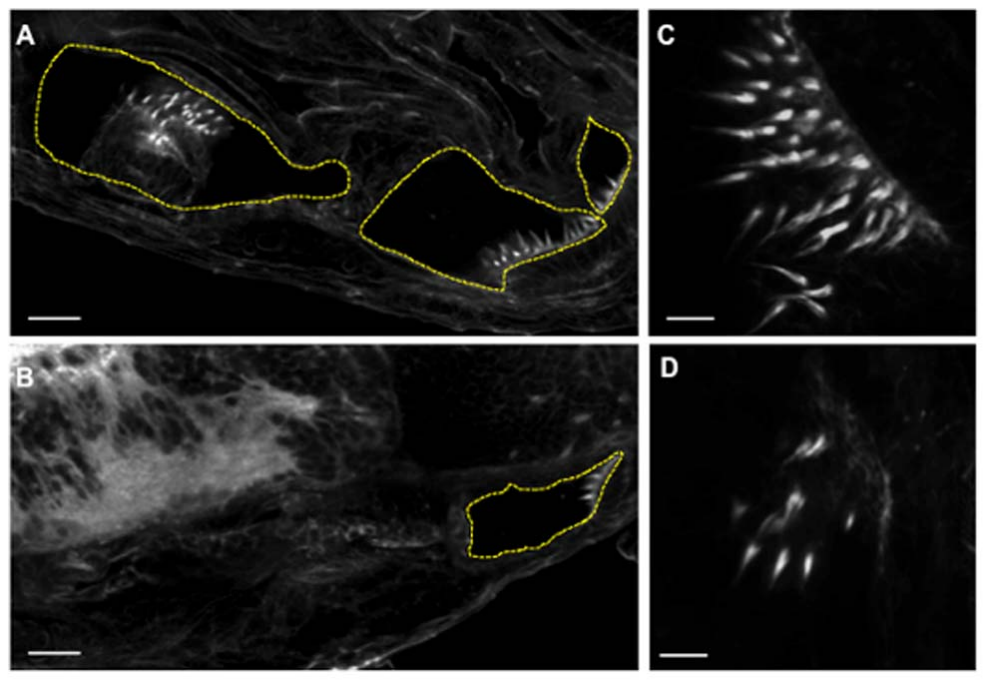
Ultra structural architecture analysis of inner ear and stereocilia performed on MO1 morphant embryos using fluorescent staining. A. Phalloidin staining of WT larvae inner ear highlighting the three sensory areas containing stereocilia. B. Phalloidin staining of 0.1: A and B: 20 µm. C and D: 5 µm.

We next investigated the structural architecture of inner ear and retinas of 0.1 mM MO1 morphant embryos using immunofluorescence. Medaka embryos have three different sensory areas in their inner ear containing hair cells necessary for hearing, movement and equilibrium (Fig. 7A and Fig. S1A). In mild morphant embryos, the sensory patches of hair cells observed in control embryos were reduced to only one area with less stereocilia with apparent normal morphology (Fig. 7 and Fig. S1A and S1B). In severe morphant embryos, recognizable stereocilia could not be detected after phalloidin staining. These results suggest that *Ol-Ush2a* participates in the embryonic development of the inner ear.

**Figure 8:**
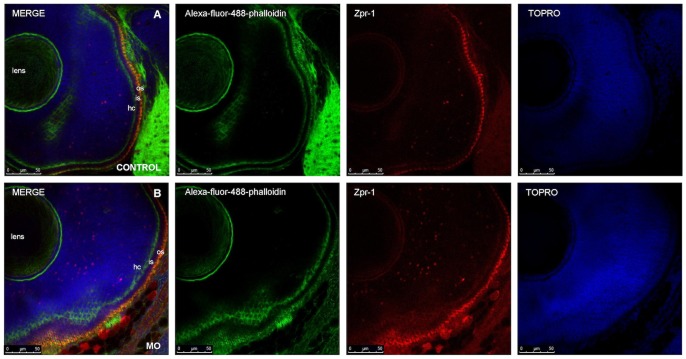
Retinal lamination observed in WT and 0.1 mM 5 dpf morphant embryos treated with PTU. Green: Alexa-fluor-488-phalloidin: actin. Blue: TOPRO: nuclei. Red: Zpr-1: double cone photoreceptors. hc: horizontal cells. os: outer segments. is: inner segments si.

The other area of *Ol-Ush2a* expression was the developing retina (Fig. 8). *Ol-Ush2a* severe morphant embryos presented disorganized small eyes; however, this effect could be due to the severity of the embryonic malformations affecting general organ structure. In order to detect more specific retinal effects, we analyzed the retinal structure of mild *Ol-Ush2a* morphant embryos that presented inner ear defects. No apparent differences were observed in retinal lamination between control and mild morphant embryos after phalloidin+anti-zpr1+TOPRO immunostaining (Fig. 8). This result indicates that inner ear development is more sensitive to depletion of *Ol-Ush2a* protein levels than retina development.

## Discussion

We have characterized the medaka fish *Ol-Ush2a* gene. *Ol-Ush2a* is 5.445amino acids in size and is encoded by a 16.5 Kb mRNA sharing the same motif arrangement with its human ortholog. Searches of genomic databases revealed that *USH2A* is remarkably conserved across phyla. It is conserved in the deuterostome lineage as well as in some (annelids and mollusks), but not all (arthropods and nematodes) protostomes [37]. Thus, *USH2A* could be an example of a gene lost in Ecdysozoa [38].

Two variants of *USH2A* are present in human and mice [5], [14]. The long variant (USH2A_iso_b, full length), participates in the formation of the ankle-link complex that anchors adjacent stereocilia to each other in the inner ear [13], [39]. In the retina, it is part of the complex participating in cargo delivery to the outer segment of vertebrate photoreceptor cells [15]. The short variant (USH2A_isoform_a), comprising only the first 5 Kb, is predicted to be a secreted, extracellular protein and was found in a subset of basement membranes [40], although functional characterization is lacking. The evolutionary analysis performed with several putative USH2A short isoform sequences from vertebrates, suggest that USH2A_isoform_a arose in eutherians and is not present in reptiles, birds, amphibians and fishes.

RT-PCR expression analysis at different medaka embryonic stages showed a strong *Ol-Ush2a* expression at early stages (0–9). These transcripts are of maternal origin, which would be in agreement with *Ol-Ush2a* expression being detected in the adult ovary. Furthermore, this early expression could explain the severe defects in complete embryonic development observed after injection of specific MOs against *Ol-Ush2a*.

At the beginning of gastrulation there is a sudden *Ol-Ush2a* expression decrease to almost undetectable levels. A low expression level is detected again from neurula stages until the somite period. After stage 30, *Ol-Ush2a* expression becomes stronger and persists in the larvae after hatching. In adult tissues, RT-PCR analysis showed that *Ol-Ush2a* was expressed in eye, but also in brain and ovary. WMISH analysis showed that the *Ol-Ush2a* expression pattern in the embryo was restricted to otoliths and retina, suggesting that USH2A might play a conserved role in the development and/or maintenance of retinal photoreceptors and cochlear hair cells. Van Wijk*et al*., (2004) [5] also found a restricted expression pattern for the long isoform_b of USH2A in humans. It was only detected in adult retina (predominantly), heart, and kidney.

Suppression of *Ol-Ush2a* using two specific MOs against the ATG starting codon site resulted in abnormal development phenotypes (small eyes and heads and shorter curved tails), similar to those phenotypes obtained in zebrafish for other ciliary proteins, encoded by genes like *RPGRIP1L*, *ZFRPGR2* or *ZFRP2* [41], [42], [43] and for the novel discovered USH gene *CIB2* [44]. Surprisingly, these malformations were not observed in zebrafish after *Ush2a* suppression using a specific MO against the splice donor site of exon 6 [23]. *Ush2a* knockdown experiments in zebrafish produced moderate levels of photoreceptor cell death in larvae. This cell death was restricted to photoreceptors and the retinas were morphologically normal. They also reported swimming and balance defects in *Ush2a* morphant larvae due to abnormal hair bundle morphology. Thus, both teleost fish present morphologically normal retinas and defects in the inner ear. Phenotype differences may be due to different penetrance levels of the morpholino effects or other technical challenges. In this sense, the use of conditional techniques to modulate the time and level of *Ol-Ush2a* knock down effect may be helpful to further develop the model and analyze the molecular mechanisms of Ush2a action. Nevertheless, one must not forget that zebrafish and medaka belong to different fish evolutive branches that are separated by an evolutive distance of about 110–200 million years ago (Mya). And so, important differences between both organisms exist that could explain the discordances observed between phenotypes [26], [45].

Additionally, *USH2A* function during mammals’ embryonic development must be redundant or practically restricted to hearing and vision since *USH2A* knockout mice and the human mutations described do not exhibit embryonic lethality.

Our data suggest that *Ol-Ush2a* is essential for medaka embryo development since loss of *Ol-Ush2a* during early development results in embryo lethality. The type of embryo defects observed in our study and in other ciliary disorders are associated with defective cell movement specifically implicated in left-right (LR) axis determination and planar cell polarity (PCP), in part due to abnormal Wnt signaling [46], [47] **,** and can result in shortened body axis, broad notochord, thin extended somites, failure of tail extension and small head and eyes [42], [48].

## Supporting Information

Figure S1 **Extended analysis of ultra structural architecture of otholits in WT (A) and morphant (B) specimens. Arrows indicate stereocilia areas.**
(TIF)
