# Supplementary figures and images for: Analysis of the Ush2a Gene in Medaka Fish (Oryzias latipes)
Source: PLoS One. 2013 Sep 23;8(9):e74995. doi: 10.1371/journal.pone.0074995 (PMC3781144; doi:10.1371/journal.pone.0074995)

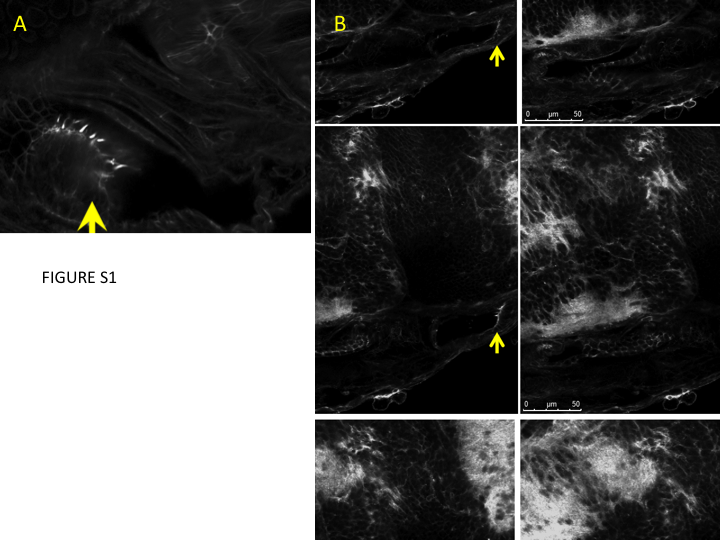

Supplement: Figure S1 — Extended analysis of ultra structural architecture of otholits in WT (A) and morphant (B) specimens. Arrows indicate stereocilia areas. (TIF) [file pone.0074995.s001.tif]
